# *N*,*N*′-Bis(4-chloro-3-fluoro­benzyl­idene)ethane-1,2-diamine

**DOI:** 10.1107/S1600536808033916

**Published:** 2008-10-22

**Authors:** Reza Kia, Hoong-Kun Fun

**Affiliations:** a X-ray Crystallography Unit, School of Physics, Universiti Sains Malaysia, 11800 USM, Penang, Malaysia

## Abstract

The asymmetric unit of the title Schiff base compound, C_16_H_12_Cl_2_F_2_N_2_, contains one half of the centrosymmetric mol­ecule. Mol­ecules related by translation along the *a* axis form stacks with short inter­molecular C⋯C distances of 3.429 (3) Å. The crystal packing also exhibits short inter­molecular Cl⋯F contacts of 3.087 (1) Å.

## Related literature

For a related structure, see Fun & Kia (2008 ▶). For general background, see: Pal *et al.* (2005 ▶); Calligaris & Randaccio (1987 ▶); Hou *et al.* (2001 ▶); Ren *et al.* (2002 ▶); Allen *et al.* (1987 ▶).
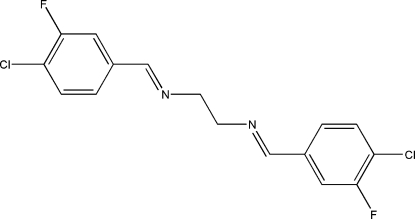


## Experimental

### 

#### Crystal data

C_16_H_12_Cl_2_F_2_N_2_
*M* *_r_* = 341.18
Monoclinic, 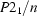

*a* = 4.6542 (1) Å
*b* = 23.1343 (6) Å
*c* = 6.9961 (2) Å
β = 107.063 (2)°
*V* = 720.12 (3) Å^3^
*Z* = 2
Mo *K*α radiation
μ = 0.47 mm^−1^
*T* = 100.0 (1) K
0.51 × 0.05 × 0.04 mm

#### Data collection

Bruker SMART APEXII CCD area-detector diffractometer
Absorption correction: multi-scan (**SADABS**; Bruker, 2005 ▶) *T*_min_ = 0.795, *T*_max_ = 0.983
17372 measured reflections
2139 independent reflections
1705 reflections with *I* > 2σ(*I*)
*R*_int_ = 0.054

#### Refinement

*R*[*F* ^2^ > 2σ(*F* ^2^)] = 0.044
*wR*(*F* ^2^) = 0.113
*S* = 1.07
2139 reflections
100 parameters
H-atom parameters constrained
Δρ_max_ = 0.99 e Å^−3^
Δρ_min_ = −0.34 e Å^−3^

### 

Data collection: *APEX2* (Bruker, 2005 ▶); cell refinement: *APEX2*; data reduction: *SAINT* (Bruker, 2005 ▶); program(s) used to solve structure: *SHELXTL* (Sheldrick, 2008 ▶); program(s) used to refine structure: *SHELXTL*; molecular graphics: *SHELXTL*; software used to prepare material for publication: *SHELXTL* and *PLATON* (Spek, 2003 ▶).

## Supplementary Material

Crystal structure: contains datablocks global, I. DOI: 10.1107/S1600536808033916/cv2465sup1.cif

Structure factors: contains datablocks I. DOI: 10.1107/S1600536808033916/cv2465Isup2.hkl

Additional supplementary materials:  crystallographic information; 3D view; checkCIF report

## Figures and Tables

**Table 1:** Selected interatomic distances (Å)

Cl1⋯F1^i^	3.087 (1)
C3⋯C6^ii^	3.429 (3)

Symmetry codes: (i) 
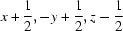
; (ii) 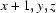.

**Fig. 1.**
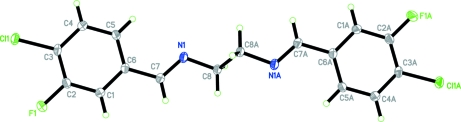
The molecular structure of (I) with atom labels and 50% probability displacement ellipsoids [symmetry code: (A) -x, -y, -z + 1].

## supplementary crystallographic information

### Comment

Schiff bases are one of most prevalent mixed-donor ligands in the field of coordination chemistry. There has been growing interest in Schiff base ligands, mainly because of their wide application in the field of biochemistry, synthesis and catalysis (Pal *et al.*, 2005; Hou *et al.*, 2001; Ren *et al.*, 2002). Many Schiff base complexes have been structurally characterized, but only a relatively small number of free Schiff bases have been characterized (Calligaris & Randaccio, 1987). As an extension of our work (Fun & Kia, 2008) on the structural characterization of Schiff base ligands, the title compound (I)  is reported here.

The molecule of the title compound, (I) (Fig. 1), lies across a crystallographic inversion centre and adopts an *E* configuration with respect to the azomethine C═N bond. The bond lengths and angles are within normal ranges (Allen *et al.*, 1987) and are comparable with those in the related structure (Fun & Kia, 2008). The planar units are parallel by symmetry but extend in opposite directions from the dimethylene bridge. The interesting feature of the crystal structure is the short intermolecular Cl···F interaction (Table 1) with the distance of 3.087 (1) Å, which is shorter than the sum of the van der Waals radii of these atoms. The molecules related by translation along the *a* axis form stacks with short intermolecular C···C distances of 3.429 (3) Å (Table 1).

### Experimental

The synthetic method has been described earlier (Fun & Kia, 2008). Single crystals suitable for *X*-ray diffraction were obtained by evaporation of an ethanol solution at room temperature.

### Refinement

All of the hydrogen atoms were positioned geometrically with C—H = 0.93 or 0.97 Å and refined in riding mode with *U*_iso_ (H) = 1.2 *U*_eq_ (C). The highest residual peak of 0.99 e. Å^-3^ is located 0.25 Å from atom H4A.

### Crystal data

**Table:**

C_16_H_12_Cl_2_F_2_N_2_	*F*(000) = 348
*M**_r_* = 341.18	*D*_x_ = 1.573 Mg m^−^^3^
Monoclinic, *P*2_1_/*n*	Mo *K*α radiation, λ = 0.71073 Å
Hall symbol: -P 2yn	Cell parameters from 3479 reflections
*a* = 4.6542 (1) Å	θ = 3.2–30.0°
*b* = 23.1343 (6) Å	µ = 0.47 mm^−^^1^
*c* = 6.9961 (2) Å	*T* = 100 K
β = 107.063 (2)°	Needle, colourless
*V* = 720.12 (3) Å^3^	0.51 × 0.05 × 0.04 mm
*Z* = 2	

### Data collection

**Table:**

Bruker SMART APEXII CCD area-detector diffractometer	2139 independent reflections
Radiation source: fine-focus sealed tube	1705 reflections with *I* > 2σ(*I*)
graphite	*R*_int_ = 0.054
φ and ω scans	θ_max_ = 30.2°, θ_min_ = 1.8°
Absorption correction: multi-scan (*SADABS*; Bruker, 2005)	*h* = −6→6
*T*_min_ = 0.795, *T*_max_ = 0.983	*k* = −32→32
17372 measured reflections	*l* = −9→9

### Refinement

**Table:**

Refinement on *F*^2^	Primary atom site location: structure-invariant direct methods
Least-squares matrix: full	Secondary atom site location: difference Fourier map
*R*[*F*^2^ > 2σ(*F*^2^)] = 0.044	Hydrogen site location: inferred from neighbouring sites
*wR*(*F*^2^) = 0.113	H-atom parameters constrained
*S* = 1.07	*w* = 1/[σ^2^(*F*_o_^2^) + (0.0465*P*)^2^ + 0.6198*P*] where *P* = (*F*_o_^2^ + 2*F*_c_^2^)/3
2139 reflections	(Δ/σ)_max_ < 0.001
100 parameters	Δρ_max_ = 0.99 e Å^−^^3^
0 restraints	Δρ_min_ = −0.34 e Å^−^^3^

### Special details

**Table:**

Experimental. The low-temperature data was collected with the Oxford Cyrosystem Cobra low-temperature attachment.
Geometry. All e.s.d.'s (except the e.s.d. in the dihedral angle between two l.s. planes) are estimated using the full covariance matrix. The cell e.s.d.'s are taken into account individually in the estimation of e.s.d.'s in distances, angles and torsion angles; correlations between e.s.d.'s in cell parameters are only used when they are defined by crystal symmetry. An approximate (isotropic) treatment of cell e.s.d.'s is used for estimating e.s.d.'s involving l.s. planes.
Refinement. Refinement of *F*^2^ against ALL reflections. The weighted *R*-factor *wR* and goodness of fit *S* are based on *F*^2^, conventional *R*-factors *R* are based on *F*, with *F* set to zero for negative *F*^2^. The threshold expression of *F*^2^ > σ(*F*^2^) is used only for calculating *R*-factors(gt) *etc*. and is not relevant to the choice of reflections for refinement. *R*-factors based on *F*^2^ are statistically about twice as large as those based on *F*, and *R*- factors based on ALL data will be even larger.

### Fractional atomic coordinates and isotropic or equivalent isotropic displacement parameters (Å^2^)

**Table:**

	*x*	*y*	*z*	*U*_iso_*/*U*_eq_	
Cl1	0.77503 (10)	0.17566 (2)	−0.30608 (7)	0.01931 (14)	
F1	0.6479 (3)	0.24323 (5)	0.0088 (2)	0.0285 (3)	
N1	0.0845 (4)	0.04180 (7)	0.2962 (2)	0.0165 (3)	
C1	0.4139 (4)	0.17032 (8)	0.1460 (3)	0.0170 (4)	
H1A	0.3865	0.1959	0.2417	0.020*	
C2	0.5544 (4)	0.18819 (8)	0.0072 (3)	0.0178 (4)	
C3	0.6002 (4)	0.15091 (8)	−0.1355 (3)	0.0162 (4)	
C4	0.5045 (4)	0.09402 (8)	−0.1403 (3)	0.0166 (4)	
H4A	0.5381	0.0684	−0.2340	0.020*	
C5	0.3581 (4)	0.07550 (8)	−0.0045 (3)	0.0158 (4)	
H5A	0.2890	0.0376	−0.0100	0.019*	
C6	0.3139 (4)	0.11314 (8)	0.1400 (3)	0.0146 (3)	
C7	0.1624 (4)	0.09406 (8)	0.2865 (3)	0.0153 (4)	
H7A	0.1227	0.1210	0.3742	0.018*	
C8	−0.0692 (4)	0.02721 (8)	0.4438 (3)	0.0154 (4)	
H8A	−0.2811	0.0208	0.3775	0.019*	
H8B	−0.0510	0.0590	0.5372	0.019*	

### Atomic displacement parameters (Å^2^)

**Table:**

	*U*^11^	*U*^22^	*U*^33^	*U*^12^	*U*^13^	*U*^23^
Cl1	0.0208 (2)	0.0198 (2)	0.0212 (2)	−0.00002 (17)	0.01230 (17)	0.00367 (18)
F1	0.0397 (7)	0.0162 (6)	0.0378 (8)	−0.0075 (5)	0.0242 (6)	−0.0041 (5)
N1	0.0177 (7)	0.0177 (8)	0.0162 (8)	−0.0006 (6)	0.0082 (6)	0.0024 (6)
C1	0.0184 (8)	0.0155 (9)	0.0192 (9)	−0.0001 (7)	0.0088 (7)	−0.0015 (7)
C2	0.0175 (8)	0.0138 (8)	0.0241 (10)	−0.0015 (6)	0.0093 (7)	0.0016 (7)
C3	0.0135 (8)	0.0185 (9)	0.0185 (9)	0.0003 (7)	0.0077 (7)	0.0048 (7)
C4	0.0168 (8)	0.0165 (9)	0.0171 (9)	0.0003 (7)	0.0061 (7)	−0.0004 (7)
C5	0.0173 (8)	0.0131 (8)	0.0185 (9)	−0.0008 (6)	0.0073 (7)	0.0017 (7)
C6	0.0139 (7)	0.0152 (8)	0.0155 (8)	0.0006 (6)	0.0057 (6)	0.0027 (7)
C7	0.0147 (8)	0.0169 (9)	0.0152 (9)	0.0003 (6)	0.0057 (7)	0.0012 (7)
C8	0.0164 (8)	0.0155 (8)	0.0165 (9)	0.0003 (6)	0.0081 (7)	0.0020 (7)

### Geometric parameters (Å, °)

**Table:**

Cl1—C3	1.7274 (19)	C4—C5	1.389 (3)
F1—C2	1.345 (2)	C4—H4A	0.9300
N1—C7	1.270 (2)	C5—C6	1.395 (3)
N1—C8	1.458 (2)	C5—H5A	0.9300
C1—C2	1.383 (3)	C6—C7	1.472 (3)
C1—C6	1.399 (3)	C7—H7A	0.9300
C1—H1A	0.9300	C8—C8^i^	1.524 (4)
C2—C3	1.383 (3)	C8—H8A	0.9700
C3—C4	1.387 (3)	C8—H8B	0.9700
			
Cl1···F1^ii^	3.087 (1)	C3···C6^iii^	3.429 (3)
			
C7—N1—C8	117.74 (17)	C4—C5—H5A	119.7
C2—C1—C6	118.90 (18)	C6—C5—H5A	119.7
C2—C1—H1A	120.5	C5—C6—C1	119.56 (17)
C6—C1—H1A	120.5	C5—C6—C7	121.36 (16)
F1—C2—C3	118.59 (17)	C1—C6—C7	119.08 (17)
F1—C2—C1	119.72 (17)	N1—C7—C6	121.70 (18)
C3—C2—C1	121.70 (17)	N1—C7—H7A	119.2
C2—C3—C4	119.55 (17)	C6—C7—H7A	119.2
C2—C3—Cl1	119.73 (14)	N1—C8—C8^i^	109.64 (18)
C4—C3—Cl1	120.72 (15)	N1—C8—H8A	109.7
C3—C4—C5	119.63 (18)	C8^i^—C8—H8A	109.7
C3—C4—H4A	120.2	N1—C8—H8B	109.7
C5—C4—H4A	120.2	C8^i^—C8—H8B	109.7
C4—C5—C6	120.65 (17)	H8A—C8—H8B	108.2
			
C6—C1—C2—F1	178.99 (16)	C4—C5—C6—C1	1.0 (3)
C6—C1—C2—C3	−0.5 (3)	C4—C5—C6—C7	−179.29 (16)
F1—C2—C3—C4	−179.76 (16)	C2—C1—C6—C5	0.2 (3)
C1—C2—C3—C4	−0.2 (3)	C2—C1—C6—C7	−179.57 (16)
F1—C2—C3—Cl1	0.1 (2)	C8—N1—C7—C6	−178.90 (15)
C1—C2—C3—Cl1	179.65 (14)	C5—C6—C7—N1	4.9 (3)
C2—C3—C4—C5	1.4 (3)	C1—C6—C7—N1	−175.37 (17)
Cl1—C3—C4—C5	−178.50 (14)	C7—N1—C8—C8^i^	−133.5 (2)
C3—C4—C5—C6	−1.7 (3)		

Symmetry codes: (i) −*x*, −*y*, −*z*+1; (ii) *x*+1/2, −*y*+1/2, *z*−1/2; (iii) *x*+1, *y*, *z*.
